# Green surgery: silencing the cry of the environment

**DOI:** 10.1097/JS9.0000000000003322

**Published:** 2025-08-22

**Authors:** Dajiang Lu, Tao Zhu

**Affiliations:** a Department of Gynecology, the People’s Hospital of Qiannan, Duyun, Guizhou, PR China; b Department of Gynecologic Oncology, Zhejiang Cancer Hospital, Hangzhou, Zhejiang, PR China

*Dear Editor,*

Environmental concerns associated with healthcare have attracted increasing global attention. The article^[1]^ by Eussen *et al*, titled “Perspectives on disposable and reusable surgical materials in laparoscopic surgery: a global survey amongst surgeons,” published in the International Journal of Surgery, deals with an important aspect of this issue. This study highlights the environmental impacts, suggesting a wider significance for the entire healthcare system. The letter aims to offer suggestions concerning future research directions and policies to address this issue.

The actual environmental impact of healthcare-related wastes may be even more severe than reported in the literature. First, although the authors included surgeons from different countries, the analysis did not sufficiently distinguish between developing and developed nations regarding the economic influences on surgical material preferences. Additionally, surgeons’ choices are often influenced by hospital or healthcare system policies rather than by personal preferences alone. Finally, single-use medical products result in environmental pollution across their entire lifecycle, including manufacturing, packaging, transportation, use, and eventual disposal. The economic incentives associated with disposable materials significantly restrict the wider adoption of reusable surgical materials, aggravating environmental issues.

To address these issues, we provide several suggestions: (1) International and national policymakers should collaborate to support developing countries in promoting economic growth and advancing healthcare systems, while establishing incentives to enable the widespread adoption of reusable and reprocessable medical devices. (2) At the governmental level, policies should actively encourage reusable surgical instruments over disposable ones. (3) At the hospital level, changes in utilization rates, cost variations, and surgeon satisfaction should be systematically tracked following the implementation of reusable surgical materials. (4) Equipment manufacturers should prioritize the development of durable, reliable, and environmentally sustainable materials. Integrating lifecycle assessments with real-world clinical data would further clarify the environmental and economic impacts. These analyses should validate the reductions in environmental pollution and the economic benefits associated with reusable materials.

These suggestions aimed to improve already excellent article better. We hope that coordinated efforts among policymakers, healthcare providers, manufacturers, and medical professionals are important to achieve harmony between medical technological advancement and environmental sustainability.

This study was adhered in accordance with the TITAN Guidelines 2025 on the declaration and use of AI in research and publication^[2]^.

## Data Availability

This article does not contain any data calculation. Not applicable.
